# Positive Microbiology in the Movies

**DOI:** 10.1111/1751-7915.70085

**Published:** 2025-09-04

**Authors:** Manuel Sánchez‐Angulo

**Affiliations:** ^1^ Alicante Institute for Health and Biomedical Research (ISABIAL) Alicante Spain; ^2^ Department of Vegetal Production and Microbiology Universidad Miguel Hernández Elche Alicante Spain

**Keywords:** education, film, popular culture, science communication

## Abstract

Microbes are essential for sustaining life in our planet. Also, we use microbes for multiple processes like food fermentation, production of antibiotics and other drugs, biofuel production, biomining and so forth. However, this essential role is scarcely represented in commercial movies. Although films are an artistic recreation of reality, we must not forget that they fix in the public's imagination the cliché that ‘microbes are bad’. In this article, some commercial movies that depict the beneficial biotechnological use of microorganisms are discussed as tools to be used in education and to engage general audiences, countering germophobia, and encourage them in the path of ‘positive microbiology’.

## Graphical abstract

How ‘Positive Microbiology’ is portrayed in commercial movies and its potential as a tool for education and engaging general audiences to counteract germaphobia. Image done with freepik.

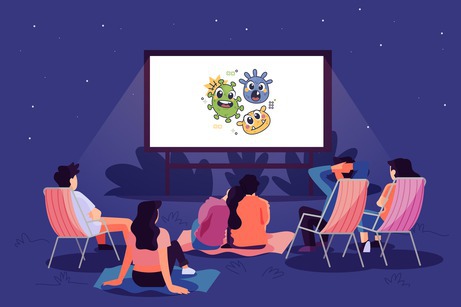

## 1 Introduction

If we put together the words ‘microbiology’ and ‘entertainment movies,’ likely we will think of hideous infectious diseases, unstoppable epidemics or even zombies. Adding the term ‘biotechnology’ often conjures images of bioweapons capable of wiping out humanity through gruesome diseases (Sánchez‐Angulo 2023). However, this skewed portrayal reflects a broader societal issue, germophobia, a socio‐cultural bias that impedes a balanced understanding of microbial activities (Oli 2023; Timmis et al. 2024). Microbes, often unfairly vilified, are essential to the functioning of the biosphere, providing enormous benefits to humanity (Timmis et al. 2019). They contribute to drug development, food production and environmental sustainability, activities that are underrepresented in popular media.

As microbiologists, we know that microbes do far more good than harm. However, movies showcasing the ‘good side’ of microbes are not very common. Cinema, as an art, appeals to the emotions, so it is a powerful element of communication that is undoubtedly attractive to the public, especially to young people. However, in this case, it should not be understood as a mere amusement, but as an opportunity to capture their attention, triggering their capacity for reflection and analysis. Previous works have shown that commercial movies or selected clips from them can be used as engaging tools in the classroom (Rose 2003; Sánchez 2011; Baños and Bosch 2015; Berlin 2016; Sánchez‐Angulo 2024). Today, the internet, streaming services and video‐sharing platforms offer convenient access to a vast array of movies and television series, ranging from old silent black‐and‐white films to modern blockbusters. The aim of this article is to discuss and curate commercial films and television series that highlight microbes' positive roles in the fields of biomedical sciences, food microbiology and environmental microbiology. Those films can be used as an educational tool for the explanation of diverse concepts—scientific, social and historical—at different audiences, from students to the general public. Only film or TV series that highlight microbes' beneficial roles in those fields were considered (Table 1). Those focused on biotechnology creating super‐pathogens or bioweapons, previously discussed in Sánchez‐Angulo (2023), were excluded. Additionally, although commercial movies do not need to be scientifically precise, they are better known to the general public than documentaries, so these last have not been considered in this article. Finally, we must remember that a featured movie is an artistical representation of reality and typically reflects the issues and concerns of the era in which it was created, rather than the time period it seeks to portray on screen. This is why a brief contextual information about the making of the movies is given.

**TABLE 1:** Non‐exhaustive alphabetical list of commercial movies related to positive microbiology and their potential use as educational tool.

Title	Year	Director	Positive microbiology aspects depicted	Other educational aspects	IMDB number
2010: The Year We Make Contact	1984	Peter Hyams	Astrobiology	Space exploration	tt0086837
A Good Year	2006	Ridley Scott	Wine fermentation		tt0401445
Arrowsmith	1931	John Ford	Phage therapy	Bioethics in clinical assays History of microbiology	tt0021622
Avatar	2009	James Cameron	Quorum sensing, bioluminescence, symbiosis	Gaia and Medea hypothesis	tt0499549
Blade Runner	1982	Ridley Scott	Synthetic Biology		tt0083658
Bottle Shock	2008	Randall Miller	Winemaking		tt0914797
Breaking the Mould	2009	Peter Hoar	Antibiotic production	Bioethics in clinical assays Scientific integrity History of microbiology	tt1434927
Dr. Ehrlich's Magic Bullet	1940	William Dieterle	Antimicrobial discovery	History of microbiology	tt0032413
Drinking Buddies	2013	Joe Swanberg	Brewery process		tt2265398
Europa Report	2013	Sebastián Cordero	Astrobiology	Space exploration	tt2051879
Fantasia	1940	Bill Roberts, Paul Satterfield (among others)	Abiogenesis, origin of life	Wine fermentation	tt0032455
French Kiss	1995	Lawrence Kasdan	Cheese varieties		tt0113117
Mad Max Beyond Thunderdome	1985	George Miller, George Ogilvie	Biomethane production		tt0089530
Medicine Man	1992	John McTiernan	Traditional fermented beverage	Imported diseases	tt0104839
Moyashimon	2007	Yūichirō Yano	Starter Cultures Strain selection	Food fermentation Microbiome Industrial microbiology	tt1143514
Nausicaä of the Valley of the Wind	1984	Hayao Miyazaki	Bioremediation	Enviromentalism	tt0087544
Red Shorgum	1988	Yimou Zhang	Traditional fermented beverage		tt0093206
Red Planet	2000	Antony Hoffman	Microbial terraformation		tt0199753
Shorts	2009	Robert Rodriguez	Microbial Bioelectricity		tt1100119
Sideways	2004	Alexander Payne	Winemaking		tt0375063
Soylent Green	1973	Richard Fleischer	Single Cell Protein	Environmental degradation Bioethics of euthanasia	tt0070723
Strange Brew	1983	Rick Moranis Dave Thomas	Brewery process		tt0086373
The Angels' Share	2012	Ken Loach	Malt fermentation		tt1924394
The Bear	2022	Christopher Storer	Food fermentation		tt14452776
The Bourne Legacy	2012	Tony Gilroy	Gene therapy	Bioethics and human experimentation	tt1194173
The Martian	2015	Ridely Scott	Composting	Space exploration	tt3659388
The Matrix	1999	The Wachowski Sisters	Single Cell Protein		tt0133093
The Story of Louis Pasteur	1936	Willian Dieterle	Vaccine development	History of microbiology	tt0028313
Vesper	2022	Kristina Buozyte Bruno Samper	Microbial Bioelectricity	Biohacking	tt20225374

*Note:* The IMDB number can be used to search the movie in the Internet Movie Database directly.

## 2 Commercial Movies and Positive Microbiology

By presenting examples from well‐known commercial movies, educators and science communicators can stimulate curiosity and open‐mindedness, counter germophobia and encourage audiences to embrace ‘positive microbiology’ (Oli 2023). This approach emphasises how safeguarding and exploiting beneficial microbes contributes to human well‐being, biosphere sustainability and even our own microbiome health (Timmis 2023). Public acceptance of these principles depends heavily on effective education, particularly by presenting microbes as allies rather than threats. Commercial movies, as teaching resources, can play a pivotal role in developing a society that is literate in microbiology. This aligns with the goals of the International Microbiology Literacy Initiative (Timmis et al. 2024), which seeks to ensure that knowledge of microbiology is accessible and relevant to the public.

Whether for the classroom or for a talk addressed to general audiences, commercial films can be used in two ways: the projection of the entire film or the use of selected short sequences (micro‐clips) with a duration of < 5 min, which concisely represent a certain microbial aspect. As indicated above, it is not common to see the positive side of microbes in films. In fact, it usually appears very briefly, even if it is connected to the main plot (e.g., wine fermentation in the romantic comedy *A Good Year*). This is why the projection of micro‐clips are more adequate for the classroom, or for general talks, than the screening of a whole film. First, I will describe the commercial movies that can be screened in their entirety and used as a teaching activity in microbiology class. The rest of the article would focus on micro‐clips that have already been used for educational purposes, detailing the reasons why it has been chosen while explaining the background of that particular film production.

## 3 The Founders of Microbiology on the Screen

There is a kind of films in which some aspects of ‘positive microbiology’ are depicted as an intrinsic part of the plot and can be suitable to be screening entirely in the classroom or even in popular sciences activities. Those movies are the biopics of the so‐called ‘founders of microbiology’ (García‐Sánchez and García‐Sánchez 2005; Kirby 2013) and were inspired by Paul de Kruif's *Microbe Hunters*. The book was published in 1926, and in those times, infectious diseases were seen as a threat that science could defeat, thanks to the development of vaccines and the discovery of the first antimicrobial substances. Nowadays, that naiveite has disappeared and the situation is quite different, as the rise of infections caused by superbugs or the emergence of new pathogens has demonstrated.

Nevertheless, the movies about Pasteur, Ehrlich or even the fictional Arrowsmith are very interesting because they can be used in the classroom to illustrate certain aspects of the history of microbiology and the ways to do scientific research in those times with the same aim as other educational initiatives that used miniatures (Guthertz 2017). If the whole movie is screened, this implies that the teacher must have previously seen the film in order to prepare a didactic guide that include a series of activities or questions to be answered by the students (e.g., What are the techniques used for bacterial identification in those times and how is it done nowadays?). Also, the activity of viewing the film should be integrated into the class syllabus. Below, there is a brief description of the main biotechnological aspects, but also other scientific and/or ethical issues, described in those biopics.

### 3.1 Phage Therapy: *Arrowsmith* (1931)

This classical film explores key aspects of the scientific vocation, including the pressure to make groundbreaking discoveries, the ‘publish or perish’ mentality and the challenges of securing research funding. It delves into the disillusionment of being preceded by other researchers in the discovery of a scientific breakthrough, the prevalence of sensationalised science in the media and the ethical dilemmas to be considered in the development of a clinical trial. Despite its dated presentation, the film remains relevant by effectively portraying the personal and professional struggles inherent in a scientific career. The book on which it is based was written at a time when substances with antibacterial power were being sought with the aim that could be used in the clinic. One of the most promising lines of research was the use of bacteriophage to tackle with infectious diseases. There is a remarkable scene that span from minute 43:00 to 53:00 that I have used in my classes to illustrate several of the aspects listed at the start of this paragraph. It goes from the euphoria of the discovery to the disappointment of learning that d'Herelle has made the same observation before. Surprisingly, a century later, phage therapy has been revived as a promising antibacterial strategy against antibiotic‐resistant bacteria (Hatfull, Dedrick, and Schooley 2022).

### 3.2 Vaccine Development: *The Story of Louis Pasteur* (1936)

This film directed by William Dieterle is told in a melodramatic tone very much to the taste of the 1930s. The axis on which the film is developed is to show the numerous efforts of Pasteur to convince the scientific community and society that diseases are caused by the infection of microorganisms. However, thanks to science, we can fight against them and so it is shown how he develops his two most famous vaccines: anthrax and rabies. Pasteur is presented to us as a visionary, a ‘mad chemist’ who is laughed at by the entire medical establishment. Despite the mockery, thanks to his moral sense, tenacity and sacrifice, he managed to carry out his research, convince the sceptics and finally triumph, being recognised and honoured by all.

### 3.3 Antimicrobial Discovery: *Dr. Ehrlich's Magic Bullet* (1940)

Paradoxically, what caused the most problems for the production of this film was precisely what Ehrlich was famous for: his discovery of arsphenamine or Salvarsan, the first chemotherapeutic agent for the treatment of an infectious disease. In the United States in 1940, there was a moral code for films and words such as ‘syphilis’ or ‘venereal disease’ were strictly forbidden. However, the president of the association responsible for the moral code himself had to admit that not naming the word ‘syphilis’ in a biographical film about the scientist who had discovered a cure would not be very logical.

The screenwriters were Norman Burnstine, Heinz Herald and John Huston. They did a very good job because the film refers to almost all the topics that Ehrlich investigated: the staining of blood cells, the improvement of Koch's staining, his work with Behring to develop the anti‐diphtheria serum, his pioneering work in the field of immunology and of course the systematic work that culminated in the development of compound 606 (Salvarsan) to treat syphilis. The film does not forget to show the other not so brilliant facets of scientific research, such as the lack of funds, the bureaucracy in applying for projects or the envy and quarrels among colleagues.

### 3.4 Antibiotic Production: *Breaking the Mould* (2009)

Unlike the previously mentioned films, this BBC production was made in more recent times. It tells the history of the development of penicillin as an antibacterial drug. It centers on the works done by Howard Walter Florey, Ernst Boris Chain and Norman Heatley in the Dunn School of Pathology during the 1938–1943 period and describes the purification problems and the development of the extraction procedure, the experiments with mice and the first and unsuccessful clinical trials with humans, the scarcity of economical funds and the ethical dilemma about patenting the process. It even describes the controversy created between Fleming and Florey, as the former tried to claim that the merit of the discovery was much more important than that of the purification.

## 4 Using Micro‐Clips on Positive Microbiology in the Classroom

Micro‐clips can be projected at any time that the teacher considers necessary throughout the teaching of a lesson or given as a part of a talk addressed to the general public. During my professional life, I have taught different microbial topics, from general microbiology to environmental microbiology. Currently, I teach an industrial microbiology course in the second year of the Biotechnology Degree in Miguel Hernandez University (Elche, Spain). I used to project the micro‐clips at the start of the class in order to improve lecture attendance, in a manner similar to the ‘microbe minute’ strategy (Feldman 2013). The screening of the micro‐clip is done without any previous presentation. Usually, the students are talking to each other so when the projection starts, they finish their chat and pay attention to it. The micro‐clips are not provided in the class website but are related with the subject to be taught that day. After the projection is finished, a brief explanation connecting the content of the film with the lecture of the day is given. Sometimes, a short debate about the subject is developed. This teaching strategy has been used from the 2014/2015‐year course to this day (2023/2024 course). The educational results of this strategy were evaluated through a voluntary and anonymous on‐line survey conducted among the students at the end of the course and after the final exam was done. Along these 10 courses, the number of students that answered the survey was 212, a 24.5% of the total of 857 students that were enrolled in the Industrial Microbiology course. Of these, 62% considered that the use of micro‐clips was very interesting and helped them to understand the topic, 35% considered that the activity was entertaining but did not improved their understanding of the topic and 3% considered that the activity was a waste of time. An additional anonymous on‐line survey was done 2 years later, when the students were in their fourth year, finishing their biotechnological careers. So, the survey recollects their memories on the micro‐clip activity. The number of answers was 137, a 23% of the total of 603 students who completed their degree. Surprisingly, 85% of the students considered that the use of micro‐clips was very interesting. In their comments, they reflect that movie sequences helped them to recall the biotechnological subject learned in the class with the pass of the time.

The following sections collect, in alphabetical order of the topic being taught, the different micro‐clips that I have used at least once in the classroom or in talks for the general public. Several of them can be found in platforms like YouTube, by performing a simple keyword search (e.g., ‘biomethane pigs Mad Max’). Others have been produced by editing a video copy of the film using Avidemux, an open‐source software. As has been indicated above, these micro‐clips are used at the start of the class, and after the projection, a related scientific fact or article is briefly discussed.

### 4.1 Astrobiology: *Europa Report* (2013)

Europa, the Jovian moon, is one of the best candidates for harbouring extraterrestrial life. It has a crust of ice and an ocean of liquid water underneath it. NASA has launched the unmanned EuropaClipper mission in October 2024 to explore this satellite and perhaps find life. In real life, sending an unmanned probe is much cheaper, safer and more feasible than sending a manned mission. However, in fiction, it is emotion that counts, not economics or safety. This a found footage film about the first manned mission to Europa. The film makes many references to the great exploratory voyages of the past, many of which were made thanks to private funding, and to the human spirit to face challenges and learn what lies beyond. The micro‐clip span from minute 55:00 to 60:00. After landing in Europa, the expedition biologist takes a spacesuit and a suitcase with scientific equipment and sets out on a solo exploration as seen in the movie poster. She manages to find a crack in the ice that allows her to take samples from Europa's ocean. Upon analysis, she finds colonial microbial forms reminiscent of the genus *Volvox* and also observes a bioluminescence phenomenon that indicates that there is something else. This movie can be used for illustrate the concept of planetary biosignatures (Malaterre et al. 2023).

### 4.2 Biomethane: *Mad Max Beyond Thunderdome* (1985)

This famous dystopic saga was created by Brian Kennedy and George Miller in 1979. They were inspired by the effects of the 1973 oil crisis in Australia. The so‐called Mad Max saga reflects a world in which oil has disappeared and modern civilization has collapsed. The few survivors are organised into warring clans that fight each other for limited resources. Mad Max (Mel Gibson) is a kind of cynical mercenary trying to weather the storm as best he can. In the third movie of the saga, Mad Max arrives in a place known as Bartertown, so called because of the numerous trade traps and which seems to be ruled by Aunty Entity (Tina Turner). However, Bartertown is actually controlled by the so‐called Master‐Blaster, a duo consisting of a dwarf and a giant. The dwarf is actually a rather gifted environmental engineer who has managed to build an entire biogas plant to produce methane from pig manure. So Bartertown is a perfect example of circular bioeconomy, showcasing sustainable waste‐to‐energy solutions like biomethane production, emphasising the role of microbes in achieving resource efficiency and ecological resilience (Ramos and Segura 2024). The pigs produce the manure which is transformed into methane which gives energy to the city which allows humans to raise more pigs. So, if someone annoyed the dwarf, all he has to do is tell his giant to turn off the gas valve to shut down the energy of all the town. In short, if the apocalypse comes, knowing how methanogens work can be very useful to us. I use a micro‐clip that span from minute 15:00 to 16:30 where Aunty Entity explain the operation of the biogas plant to Mad Max as the introduction for the lesson on biofuels.

### 4.3 Bioremediation: *Nausicaä of the Valley of the Wind* (1984)

The movie takes place 1000 years after a war that devastated the planet, contaminating a large part of its surface and generating the appearance of huge forests of toxic fungi and giant insects. Humans try to survive in isolated communities that inhabit the few uncontaminated areas and reminiscent of ancient medieval kingdoms, albeit with more advanced technology. These kingdoms are in constant struggle for scarce resources. Naussicaä is the princess of the so‐called Valley of the Wind, and besides being a nature lover, she is an intrepid explorer and a self‐taught mycologist. She has spent her whole life studying mushroom forests and trying to understand their life cycle. In doing so, she will discover that the fungi are bioremediating the environment, removing pollution from the soil and allowing plants and animal life to grow. The problem is that his discoveries come just as the Valley of the Wind is being invaded by a neighbouring kingdom.

Miyazaki was inspired by the works of Ursula K. Le Guin, Tolkien, Asimov and of course Homer, to create the world of Nausicaä. However, there was a specific event that originated the idea of the contaminated world and this was known as the Minamata Bay incident, in which mercury contamination caused the massive poisoning of the local population. The Chisso Company was dumping mercury sulfate into the bay over several years. The mercury sulfate accumulated in the sediments and was metabolised there by sulfate‐reducing bacteria that transformed it into methyl mercury. In other words, a phenomenon of bio‐magnification of a pollutant was triggered, since methyl mercury accumulates in the food chain, especially in the fatty tissue of fish. The consumption of these fish caused many people to suffer from a neurological disease known as Minamata disease (Ekino et al. 2007).

### 4.4 Cheese Varieties: *French Kiss* (1995)

Cheese is a staple food very well‐known, but its production has been scarcely represented in commercial movies. Probably this romantic comedy has the most famous scene about the numerous varieties of cheese. A just awaken Kevin Kline observes an enthusiastic Meg Ryan while eating a breakfast consisting of a dish assorted with different French cheeses and saying with satisfaction: ‘*Did you know that there are 452 official government cheeses in this country? Don't you think that's incredible, to come up with 452 ways of classifying what is basically a bacterial process?*’. I use this micro‐clip, that span from minute 58:15 to 60:15 of the movie, as the introduction for the lesson on lactic fermentation products.

### 4.5 Compost and Agriculture: *The Martian* (2015)

The chief role of microbes in this movie is their enabling to grow healthy and beautiful potatoes in Mars. Matt Damon puts into practice the Louren Bass' principle ‘Everything is everywhere, but the environment selects’ (de Wit and Bouvier 2006). Human faeces contain microbes able to interact with plants and colonise the rhizosphere. Furthermore, it is possible to grow various plants rooted in simulated Martian soil (Wamelink et al. 2014). So, the idea of using manure as a fertiliser on Mars is not at all far‐fetched, but it may have been better to compost it first. Another situation that can be criticized from a microbial point of view will be happen later. At one point there is a breach in the greenhouse structure; it undergoes a sudden decompression and it suddenly freezes. In the film, we are told this means that supply of potatoes has run out, not because the plants could not be replenished, but because the microbes froze and died. A simple glance to a laboratory freezer rules out that possibility. The complete sequence of planting and growing potatoes in Mars is very long to be used as a micro‐clip, but in the internet is very easy to find edited clips from the movie that shows the whole process in < 5 min.

### 4.6 Food Fermentation: *The Bear* (2022)

This recent television series tells the history of Carmy (Jeremy Allen White), an award‐winning chef who returns to his hometown to manage a fast‐food restaurant previously owned by his deceased brother. As expected he finds a filthy place immerse in chaos but his aim is transforming the premises in a fine dining restaurant. One of his first decisions was to train the restaurant baker to be the pastry chef, starting with the basics: teaching him how to properly ferment bread, and from there, to make other types of pastries and cakes. In episode 5 from season 1, Carmy leaves him a very special book: *The Noma Guide to Fermentation* written by David Zilber and René Redzepi (the former was the fermentation master and the latter the chef of the Noma restaurant). The book is an authentic manual for making any type of food fermentation, from fruits and vegetables with lactic bacteria, to vinegar of any kind, to making miso and even garum, the famous Ancient Roman sauce. The book also gives very basic but very precise notions of microbiology, pH chemistry and, above all, food safety to avoid contamination and possible health risks. I use that particular movie‐clip to introduce the initial lesson on food fermentation.

### 4.7 Gene Therapy and Bioethics: *The Bourne Legacy* (2012)

In this action‐packed movie, Jeremy Renner gives life to a super‐agent that has undergone a genetic doping treatment by using a virus. Notably, the ‘mad scientist’ in this movie is Rachel Weisz, a female biochemist that complains in this way about her role.

I was there for the science. We were all there for…for science! And I know you don't care, but I made a huge sacrifice. I couldn't publish, I couldn't conference, I couldn't tell a single person what it was I did! But I thought I was…I thought I was helping my country and I know that…

This movie‐clip that span from minute 71:00 to 74:00 can be used to discuss several bioethical issues like the patient consent, the responsibility of the scientists about their work and the genetic alteration of human beings. In my case, I have used this particular micro‐clip to introduce a debate in class on genetic doping in sports (Cantelmo et al. 2020).

### 4.8 Malt Fermentation: *The Angels' Share* (2012)

This comedy‐drama is about a group of Scottish misfits that discover the world of whisky as a way to redemption. The sequence of the distillery tour that span from minute 30:15 to 34:40 is quite interesting, and I often use it in the introduction of the lesson dedicated to the equipment in industrial microbiology. In the sequence, the whole process of making pure malt whiskey is explained, from the fermentation vats of malted barley, through the distillery and finally the maturation and aging of whiskey in oak barrels. At the end of the visit, there is a whisky tasting in the cellars, where the guide explains that in the aging process, a part of the liquor contained in the barrels is always lost through evaporation. This lost whiskey is what is known as ‘the angels' share’. After the visit, the main character discovers that he has a special gift for identifying flavours and could become a good taster. However, his past as a juvenile delinquent still haunts him, so with the help of his community service colleagues, he has to find a way to leave his old life behind and start over with his wife and son.

### 4.9 Microbial Terraformation: *Red Planet* (2000)

The terraforming of other planets is a recurring theme in science fiction films such as *Aliens* or *Total Recall*. In this movie, the target of terraforming is the planet Mars. At the very beginning, we are shown beautiful images of Mars while a voice‐over tells us that in the year 2050, the Earth's resources are running out and mankind is looking for another place to live. For several years, spacecraft containing unicellular algae have been sent to make the atmosphere of Mars breathable. In parallel, we see how the reddish Martian surface is progressively changing to a green colour. At a given moment, the greenness stops and degrades and the planet turns reddish again. The explanation describes that something is destroying the algae and that the oxygen levels in the Martian atmosphere are dropping. Therefore, it has been decided to send a scientific exploration mission to find out what is causing the algae to disappear. This opening is the best part of the film, and I have used this sequence as an introduction to the lecture on microbial photosynthesis. From that point on, the plot turns into a collection of absurdities and clichés.

### 4.10 Quorum Sensing and Symbiosis: *Avatar* (2009)

I have used movie‐clips from this famous movie in environmental microbiology classes and in talks addressed to the general public with the aim to introduce and explain the well‐known symbiosis between the bioluminescent bacteria *Vibrio fischeri* and the squid *Euprymna scolopes* (Visick, Stabb, and Ruby 2021), but the movie can be used to discuss other scientific concepts. After its premiere, numerous articles appeared in the press and on the web about the different scientific aspects represented in the movie. The main biological aspect of *Avatar* is that the planet Pandora is a recreation of the Gaia hypothesis formulated by James Lovelock in 1972. James Cameron imagined that the entire biosphere of Pandora can be interconnected with each other. Every ‘vertebrate’ shows a kind of bio‐USB that allows the physical connection. Also, it can be used to connect with the plants through a kind of fluorescent mycorrhizae. At first glance, each living being behaves in a Darwinian way—eat or be eaten, reproduce and leave offspring—but when the time comes, the connection is so effective that Pandora's biosphere behaves like a mega‐superorganism capable of acting against external threats such as Earthlings. Incidentally, the Gaia hypothesis is opposed to the Medea hypothesis, which considers life to be a self‐destructive and suicidal superorganism.

In the lecture about quorum sensing and symbiosis, I like to use as introduction a micro‐clip in which Dr. Augustine (Sigourney Weaver) is seriously injured. The scene spans from minute 114:40 to 115:40, in which the Na'vi take her body to the Tree of Souls and attempt to transfer her consciousness to the alien avatar. The entire tribe form into a circle, unite through their bio‐USBs and in turn unite with the Tree of Souls through fluorescent mycorrhizae that are in contact whit their bodies. Gradually, a series of pulsating bioluminescence waves begin to appear as their bonding becomes tighter.

### 4.11 Single Cell Protein (SCP): *Soylent Green* (1973) and *The Matrix* (1999)

A current topic in industrial microbiology is the production of food from animal cell cultures in the laboratory, a technology popularly known as in vitro meat. This technology is an evolution of the concept of SCP, aimed at producing food from microorganisms (Li et al. 2024). To introduce this topic in my classes, I have used movie‐clips from two movies: *Soylent Green* and *The Matrix*. A curious note is that both movies are science fiction dystopias. *Soylent Green* is part of the ‘Charlton Heston's dystopian trilogy’, the other two being *Planet of the Apes* (1968) and *The Omega Man* (1971). This film is based on Harry Harrison's novel *Make Room! Make Room!*, about an overpopulated Earth. Metro Goldwin Mayer bought the rights and commissioned screenwriter Stanley R. Greenberg to make some changes. One of those was the creation of the ‘Soylent Green’, the planktonic microalgae‐based crackers used as food rations. Incidentally, Greenberg took the opportunity to introduce a main plot totally different from that of the novel. Set in the year 2022, the movie has become a Sci‐Fi classic because it touches on many topics that are still relevant today, such as overpopulation, environmental pollution, objectification of woman, euthanasia, management of information and so forth. It is also famous for being the last film starring actor Edward G. Robinson. The micro‐clip that I used from this movie spans from minute 3:00 to 3:30; a commercial advertisement about the different Soylent varieties is running in a TV monitor.

In the case of *The Matrix*, one of the most iconic science fiction movies of all time very well‐known by the general public, the film portrays a dystopian future where most humans are enslaved by machines, with only a small resistance fighting back. The micro‐clip that I use spans from minute 65:00 to 66:13. After the main character, Neo (Keanu Reeves), is freed, he is served a meal consisting of a disgusting white porridge. A crew member explains, ‘*It's a single cell protein combined with synthetic aminos, vitamins and minerals. Everything the body needs*.’ While the SCP in this future appears unappetising, modern SCP products, like mycoprotein as in Qorn or bacteria in Uniprotein, are far more palatable (Souza Filho 2022). In class, I show this sequence before explaining the topic, as the familiarity of the movie immediately captures the students' attention. Afterwards, I ask them if they would eat a meal made entirely from microbes. This sparks a lively discussion, followed by a revelation: they probably already have. I use to show them pictures of various microbe‐derived foods that can be found in the supermarket's aisles.

### 4.12 Starter Cultures: *Moyashimon. Tales of Agriculture* (2007)

This 2004 manga written and illustrated by Masayuki Ishikawa was adapted as a television series in 2007. It tells the story of Tadayasu Sawaki, a first‐year college student at an agricultural university, who has a unique ability to see and communicate with microorganisms. Guide by Keizō Itsuki, his microbiology professor, he will develop the full potential of his gift. Several fermentations are explained with great detail, like the ones to produce sake, soy sauce, miso and even French wine. Also, the role of the microbiota is explained. It is relatively easy to find some episodes in the different video platforms in the web. At the end of each episode, a post‐credit scene, called ‘Microbe Theatre’, is shown. In that end tag, a particular microbiological concept is explained in less than a minute, so they are ideal micro‐clips. In my classes, I have used two particular scenes: one from the episode 4 of the first season, where the concept of selected yeast to produce starter cultures for beverage fermentation is explained, and the other from the episode 6 of the second season that explains the methods of generation of mutants in microbes of interest by using UV irradiation and heat‐shock.

### 4.13 Synthetic Biology: *Blade Runner* (1982)

We have passed 2019, and there are neither flying cars, nor have we travelled to the stars, nor are there C‐Rays glowing in the dark, and of course, there are no ‘replicants’. However, despite the fact that most of its predictions have not come true, *Blade Runner* remains a warning of a possible future. If there is one scientific aspect in which this movie got it right, it is the depiction of the incredible potential of biotechnology. The film talks about organs culture in the lab and artificial living beings. A sequence considered minor, but nevertheless very significant, is at minute 46, when Deckard (Harrison Ford) needs an analysis of a snake scale. Under the electronic microscope, a serial number is observed on the surface of the scale identifying the manufacturer. Perhaps we have not reached that point, but we do have a designed living being with watermarks, as is the case of Craig Venter's *Mycoplasma laboratorium* (Gibson et al. 2010).

### 4.14 Wine Fermentation: *A Good Year* (2006)

I used a micro‐clip, spanning from minute 53:24 to 55:00, as the introduction to a lesson on wine fermentation. After the success of *Gladiator*, Ridley Scott and Russel Crowe teamed up again for this romantic comedy set in the French countryside. The micro‐clip shows the inside of a wine cellar where a child observes the cap of grape skins formed at the top of a wine cask. As the grapes ferment, the surface bubbles actively. The child is questioned by his uncle about what he sees on that surface, and because he has been taught by the wine maker, he correctly answers ‘*Fervere—it's a Latin term. It means ‘to boil’. The native yeasts in your cellar are converting the sugar inside the grapes to alcohol. The release of carbon dioxide gas is what causes the bubbling of it*’.

### 4.15 Winemaking: *Bottle Shock* (2008)

I used the scene that spans from minute 49:20 until 50:15 to show the importance of the grapes and the *terroir* in the making of high‐quality wine, but this movie can also be used for the explanation of other concepts in winemaking, including the economic and cultural ones. For wine enthusiasts, the expression ‘Judgment of Paris’ has an additional meaning besides the mythological one. In 1976, a blind tasting contest was held in Paris between French and American white and red wines. Against all odds, an American wine won in both categories: in whites, the ‘Chateau Montelena’ and in reds the ‘Stag's Leap Wine Cellars’, both from 1973 and produced in Napa Valley. Since then, the world of wine has never been the same. The commercial and cultural impact was so important that a couple of bottles of these wines are exhibited in the American History Museum of the Smithsonian Institution.

Although the film does not mention the microbiological aspects of wine fermentation, it does about the different stages necessary to make a good wine like the kind of soil to grow the grapes, the water stress conditions to obtain the correct sugar concentration, the racking process to clarify the wine and the importance of pressing the grapes avoiding the crushing of the seeds. In fact, the title refers to the instability that occurs in wines when they are transported and shaken (hence the idea of letting a wine rest when you bring it back from a trip). In the case of white wines, a phenomenon called ‘pinking’ can occur, which is due to the oxidation of polyphenols. If it is very intense, a brownish colour may develop and the wine will have a metallic taste, so it is usually discarded. However, the film invents an explanation based on a reversible enzymatic phenomenon due to the extreme care taken by the winemaker to maintain the reduction conditions.

## 5 Positive Microbiology for Children

If it is hard to tackle germophobia in the high‐school or undergraduate education, it is harder in children's primary education levels. Since microbes are invisible to the naked eye, teaching children about microbial activities requires creative strategies to bridge the gap between observation and understanding (Timmis, Timmis, and Jebok 2020; Timmis et al. 2024). Some famous commercial films can be useful for teaching positive microbiology to children. My experience with these films does not come from my professional role as a teacher or science communicator, but rather from my personal role as a father. I have used them to educate my daughters and help them understand the nature of my work in microbiology.

### 5.1 Origin of Life: *Fantasia* (1940)

The segment *The Rite of Spring* was directed by Bill Roberts and Paul Satterfield, with animators such as Wolfgang Reitherman and Joshua Meador. Igor Stravinsky's music was adapted by Leopold Stokowski, who rearranged the pieces and excluded complex sections. Although the sequence is spectacular, Stravinsky harshly criticised both the arrangements and the performance by the Philadelphia Orchestra, calling it ‘execrable.’

The narrative of this segment addresses the origin of Earth, the emergence of life and its evolution up to the extinction of the dinosaurs. In the microbiological context, the sequence reflects the scientific ideas of the time, particularly the abiogenesis hypothesis proposed by Oparin and Haldane, based on the ‘primordial soup.’ This theory suggested that volcanic energy and lightning generated organic compounds that eventually gave rise to life. The animation illustrates this process: lava and waves generate steam, followed by the appearance of unicellular organisms like a euglena swimming with its flagellum and evading an amoeba. These simple forms multiply and evolve into multicellular organisms. In just a few minutes, the segment impressively condenses 3.5 billion years of evolution, showcasing a remarkable depiction for its time.

### 5.2 The Problem of Spatial Scale: *Horton Hears a Who!* (1970, 2008)

The story of Horton, a kind‐hearted elephant who hears a faint cry for help coming from a tiny speck of dust. Realising the speck contains a miniature world called Whoville, inhabited by microscopic beings called Whos, Horton vows to protect them. He tries to tell the other jungle animals that an infinite number of tiny beings live on that tiny speck of dust, but no one believes him, and they label him as crazy. Meanwhile, Mayor Ned from Whovile, also tries to convince his fellow citizens that beyond the clouds covering their world, there is another much larger reality. However, they too think their mayor is a delusional dreamer filled with fantasies.

Seuss was inspired by a personal experience to write the story of Horton. During World War II, he worked in the U.S. Army's propaganda services and naturally developed strong anti‐Japanese sentiments. However, after the war ended, he visited Japan in 1953, and upon witnessing the damage suffered by the populations of Hiroshima and Nagasaki, his feelings changed. This is why Horton's mantra is ‘A person's a person, no matter how small’.

Horton and the Whos conveys lessons about equality and fair treatment while introducing children to key scientific concepts. It illustrates the problem of spatial scale, showing how tiny microbes can still have a significant impact, emphasises the importance of instruments for exploring the cosmos and reflects the challenges scientists face in explaining the complexity of the world beyond what is immediately visible.

## 6 Discussion

Commercial movies could serve as valuable educational tools for teaching complex concepts, including those related to microbiology and microbial biotechnology. A diverse array of microbial themes is represented in popular films, making them suitable not only for life science students but also for those studying arts or history (Gurnon, Voss‐Andreade, and Stanley 2013; Oren 2015). While microbes are often portrayed negatively as agents of disease, epidemics or bioweapons (Sánchez‐Angulo 2023), the works presented here show that there are some films that highlight their beneficial roles, such as wine fermentation, biomethane production or microbial bioremediation. These examples, whether integral to the plot or featured briefly, showcase the positive impact of microbes on society. We must not forget that movies also reflect the cultural issues and concerns of the era and situations in which they were made, rather than the historical periods they depict. This may result in limitations in the representation of some microbial activities that may be well known. For example, despite beer's ubiquity in culture, there are almost no quality films dedicated to its brewing process, in stark contrast to the many films celebrating the elegance of winemaking. Additionally, of the 29 films listed in Table 1, 13 are science‐fiction movies, indicating that filmmakers have seen microbes' capacities as promising technologies, some of which have delivered on their promises, while others are still far from doing so. In consequence, educators must approach these films with preparation and critical analysis. It is essential to view the film beforehand, design educational activities like questionnaires and identify errors or misconceptions for correction. This preparation ensures that films are used effectively to meet educational objectives.

Table 1 lists other movies that have not been discussed in this work but offer potential for further integrating microbial biotechnology and positive microbiology into education. By using both popular and lesser‐known films, educators can promote microbial literacy and correct germophobic biases. These small but mighty organisms will undoubtedly continue to inspire filmmakers, providing opportunities to foster curiosity and appreciation for their role in life and technology.

## Author Contributions

**Manuel Sánchez‐Angulo:** conceptualization, investigation, writing – review and editing.

## Conflicts of Interest

The author declares no conflicts of interest.

## Data Availability

The data that support the findings of this study are available from the corresponding author upon reasonable request.
